# Entropion in a Dog. (Dianour’s Operation)

**Published:** 1890-03

**Authors:** Gerald G. Griffin

**Affiliations:** Fort Reno, I. T.


### ENTROPION IN A DOG. (DIANOUR'S OPERATION).

By Gerald G. Griffin, D.V.S.
Fort Reno, I. T.

I was requested to examine a valuable pointer dog by the owner, who stated the animal was slowly becoming blind. On superficial examination I found the subject 4 years old, in good health (temperature, pulse and respiration normal ; appetite good) with the exception of the right eye, which appeared considerably inflamed and showing a profuse watery discharge. The owner stated the animal was apparently all right until about six months previous, when it contracted a severe cold which "seemed to settle in his eyes." Since that time it had difficulty in "fielding," and, when worked, the eyes seemed to grow worse.

Having made a thorough examination I found that the lower eyelid was completely inverted, so much so, indeed, that the cilia was entirely hidden from view and left only a small horizontal slit, through which but a portion of the cornea was visible. On exploration with ophthalmoscope I found the cornea so opaque that evidently no light passed through. I observed a purulent collection between anterior portion of the sclerotica and eyelids ; the left eye appeared slightly inflamed and showed a scant, watery discharge. This was evidently sympathetic. I came to the conclusion that an operation would have to be performed and so advised the owner, who was afraid it would spoil the appearance of the animal. I then advised him to use astringent washes and suggested epilation. The latter he objected to. Washes were persisted in for two weeks but without favorable result, when the owner decided on an operation. The patient was placed under ether, integument of lower eyelid cut through parallel to its edge and about four lines from the same. The incision reached the cartilage only. On intermarginal edge the lid was split between the cartilage and muscle until the first incision was met, at right angles to it, thus forming a bridge of lid containing cilia and their roots. Another incision was made parallel with the first about two lines below it, leaving a riband of skin between the two, which was dissected up, leaving it attached at both ends. The bridge of lid margin was then drawn down and over the riband of skin and fastened by silk suture to lower border of lowest incision, while the integument riband was drawn up beneath and stitched to intermarginal edge. The sutures were taken out in five days, when the wound was found healing by first intention. The cornea gradually cleared up, the left eye became normal, and in ten days after the operation the animal was discharged.

Entropion was probably brought on by blepharitis which was neglected. I used atropine twice on cornea after operation.

